# Exosomes in Colorectal Cancer: From Physiology to Clinical Applications

**DOI:** 10.3390/ijms24054382

**Published:** 2023-02-23

**Authors:** Stefan Titu, Vlad Alexandru Gata, Roxana Maria Decea, Teodora Mocan, Constantin Dina, Alexandru Irimie, Cosmin Ioan Lisencu

**Affiliations:** 1 Faculty of Medicine, “Iuliu Hatieganu” University of Medicine and Pharmacy, 400126 Cluj-Napoca, Romania; 2 Department of Surgical Oncology, The Oncology Institute “Prof. Dr. Ion Chiricuta” Cluj-Napoca, 400015 Cluj-Napoca, Romania; 3 Department of Oncological Surgery and Gynecological Oncology, “Iuliu Hațieganu” University of Medicine and Pharmacy, 400012 Cluj-Napoca, Romania; 4 Department of Physiology, Faculty of Medicine, “Iuliu Hațieganu” University of Medicine and Pharmacy, 400012 Cluj-Napoca, Romania; 5 Nanomedicine Department, Regional Institute of Gastroenterology and Hepatology, 400126 Cluj-Napoca, Romania; 6 Faculty of Medicine, Ovidius University, 900527 Constanta, Romania

**Keywords:** exosome, cancer, colorectal cancer, exosomal miRNA, lncRNA

## Abstract

Exosomes are nanosized vesicles that have been found to be involved in many diseases. Exosomes can mediate communication between cells in a variety of ways. Certain types of mediators derived from cancer cells can play a crucial role in the development of this pathology, promoting tumor growth, invasion, metastasis, angiogenesis, and immunomodulation. Exosomes in the bloodstream show promise as a future tool for detecting cancer at an early stage. The sensitivity and specificity of clinical exosome biomarkers need to be enhanced. Knowledge of exosomes is not only important for understanding the significance of cancer progression but also for providing clinicians with useful information for the diagnosis, treatment, and discovery of methods to prevent cancer from recurring. The widespread adoption of diagnostic tools based on exosomes may revolutionize cancer diagnosis and treatment. Tumor metastasis, chemoresistance, and immunity are all aided by exosomes. A potential new approach to cancer therapy involves preventing metastasis by inhibiting miRNA intracellular signaling and blocking the formation of pre-metastatic niches. For colorectal patients, exosomes represent a promising area of investigation for improving the diagnosis, treatment, and management. Reported data demonstrate that the serum expression level of certain exosomal miRNA is significantly higher in primary colorectal cancer patients. The present review discusses mechanisms and clinical implications of exosomes in colorectal cancer.

## 1. Exosomes

Exosomes are nanosized vesicles that have been found to be involved in many diseases. They are secreted by various cell types upon the fusion of multivesicular bodies and the plasma membrane [1]. Exosomes are typically 40–150 nm in diameter and carry nucleic acids, proteins, lipids, and metabolites [2]. Exosomes eventually generate multivesicular endosomes (MVEs) that are secreted into the extracellular space to travel to other cells [3]. Originally, when released from cells, exosomes were considered cellular garbage collectors following cell degradation or loss of cellular homeostasis without playing an important role in the surrounding body cells. However, more recent findings have showed that they mediate cell–cell communication, being loaded with proteins, lipids and nucleic acids that are delivered to target cells, and they are able to alter the biological behavior of the recipient cells [4]. Various surface molecules are shown to be responsible for the interaction between extracellular vesicles and recipient cells for their uptake. After they bind to the target cell, several processes may occur, receptor–ligand interaction, endocytosis and/or phagocytosis or membrane fusion and further load delivery into the cytosol and the subsequent change in the physiological state of the recipient cell [4]. There have been several studies where all membrane-bound vesicles are largely cited as extracellular vesicles and not particularly referred to as exosomes, microvesicles or other subtypes. Nevertheless, it is necessary to clearly distinguish exosomes from other extracellular vesicles in order to comprehend their action and compare various study results [5]. The biogenesis of exosomes involves their origin in endosomes, and they exhibit membrane protein expression profiles involved in membrane transport and fusion such as Rab GTPases, annexins and flotillin, components of the ESCRT complex, integrins and tetraspanins, including CD9, CD63, and CD81 [6].

One of the basic functions of exosomes is the elimination of excessive proteins or undesirable molecules from the cell, but they are important mediators of intercellular communication and are involved in various pathways being biologically active vesicles released into the extracellular environment [1].

**Figure 1:**
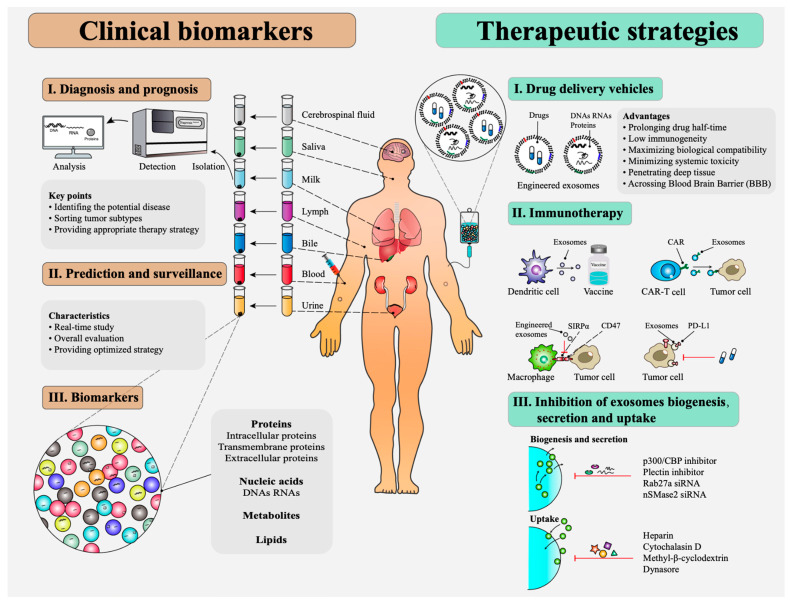
Key applications of exosomes. Reprinted with permission from Zhu, L. et al., 2000 [7].

Exosome engineering through genetic and chemical methods for targeted drug delivery may help increase their therapeutic applicability as clinical biomarkers [7]. There are still a lot of aspects to be considered for the design of new cancer treatment strategies, but exosomes exhibit great potential in precision cancer medicine. Figure 1 is broadly depicting all clinical applications that exosomes may have.

As exosomes have proved their key role in cancer processes, there are three main research areas with clear participation in cancer progression: exosomes can modulate host immune response and induce immune tolerance; exosome crosstalk with the tumor microenvironment promotes tumor growth and progression; and their significant role in metastasis [2].

More exosomes are produced and released by cancer cells than by healthy ones, and the molecules found in exosomes released by tumor cells are very different from those found in healthy ones. Recent studies have shown that there are substantial differences between colorectal cancer (CRC) patients and healthy controls in the levels of certain microRNAs (miRNAs), long non-coding RNA (lncRNAs), and proteins found in exosomes isolated from blood (NCs). Some research suggests that these exosomal molecules can serve as markers for colorectal cancer.

## 2. The Role of Exosomes in Human Disease in General and Cancer in Particular

### 2.1. Exosome Modulation of the Immune System

There have been various studies on the role exosomes play in immune regulation, with a more recent one focusing on how exosomes regulate the immune response [8].

It has been demonstrated that human Epstein–Barr virus-infected B cells secrete exosomes carrying Major Histocompatibility Complex (MHC) classes I and II, thus indicating their potential implication in the modulation of immune responses [9]. This finding has triggered numerous other studies that have confirmed that exosomes secreted by antigen-presenting cells, for example, DCs, express class I, class II MHC, adhesion, and co-stimulatory molecules. Such features allow exosomes to directly activate CD8+ and CD4+ T-cells and induce a strong immune response [1]. Peptide-pulsed dendritic cells release immunogenic exosomes and stimulate a strong CD8+ T-cell-dependent anti-tumor immune response [10].

Exosomes derived from cancer cells express tumor antigens able to activate dendritic cells, therefore determining immune priming and triggering a specific cytotoxic response superior to the immunogenicity of tumor cell lysates or soluble antigens in vaccines [11]. It has been shown that one intraperitoneal injection of tumor peptide-loaded dendritic cell-derived exosomes can trigger a very powerful immune response that could lead to tumor growth delay or tumor rejection [12]. While this could be attributed to high antigen density, it is also due to the presence of heat shock proteins as seen in the case of exosomes produced by melanoma cells [13,14].

Exosomes trigger immune response suppression, leading to the low immunogenicity observed in several studies. Exosomes derived from cancer cells can suppress natural killer cells by downregulating NKG2D expression [15].

Dendritic cell maturation is impaired in vivo by tumor cell-derived exosomes, therefore leading to immunosuppression. Breast cancer cell-derived exosomes are internalized by bone marrow myeloid precursors, impairing dendritic cell differentiation by promoting IL6 overexpression and Stat3 phosphorylation [16]. Subsequent research showed that bone marrow precursor cells isolated from an IL6 knockout (KO) model can differentiate into dendritic cells following treatment with exosomes derived from cancer cells. Altogether, these study results indicate the immunosuppressive potential of tumor cell-derived exosomes via NK and DC modulation. Still, not all findings can identify the effector molecules initiating the modulation of the immune response [2].

### 2.2. Exosomes and Cancer

Cancer progression is determined by the crosstalk between cancer cells and the neighboring cells. This type of cell-to-cell communication is based on dynamic information exchange, inducing a pro-tumor microenvironment where carcinogenesis occurs and the immune response is modulated in order to promote tumor progression and survival [1].

Exosomes are essential components of the intercellular microenvironment, acting as regulators of cell-to-cell communication. It has been widely demonstrated that exosomes can induce phenotypic changes in neighboring cells through the activation of specific cell-signaling pathways leading to cancer progression [17].

Extensive studies have been carried out on intracellular communication, mainly during tumor development. Exosome-associated RNAs, miRNAs, proteins, DNAs, and even metabolites are able to determine changes in the outcome of recipient cells via autocrine and paracrine signaling mechanisms. Exosomal proteins are able to modulate the outcome of exosome-secreting cells through autocrine signaling. More specifically, chronic myeloid leukemia-derived exosomes contain TGFβ1, a cytokine that binds to the TGFβ1 receptor in leukemia cells and further promotes tumor growth by the activation of ERK, AKT and anti-apoptotic pathways in producer cells [5].

Some of their characteristics make exosomes superior to other extracellular vesicles for use as therapeutic agents, such as their stability in vivo and in vitro, bioavailability, good distribution into the surrounding body fluids, their ability to successfully cross the blood–brain barrier, good tolerance and regulation of gene expression by transferring miRNA and siRNA into target cells. All these features indicate their potential role in anti-cancer vaccines as well as natural liposomes for targeted delivery with various options for novel cancer therapies [1].

Mitochondrial DNA components were detected in exosomes, resulting from the culture supernatant of myoblasts and chromosomal DNA (vide infra). Chromosomal DNA was identified in cell culture supernatant in both human and mouse biological fluids, such as blood, seminal fluid, and urine. DNA-loaded exosomes could enhance DNA stability after it leaves the cell [18]. Such findings promote the use of exosomes as novel biomarkers in liquid biopsies, assisting the diagnosis and monitoring of cancer patients [19]. Blood plasma exosomes containing circulating DNA are complex agents in cancer therapies, isolating cancer-specific DNA for circulating cancer cell-derived exosomes [20].

Fibroblast-derived exosomes were shown to stimulate the protrusion of breast cancer cells (BCC) as well as their motility and metastasis dependent on tetraspanins, namely Cd81, which are common EV-associated markers. A study on a mouse model showed that tumor exosomes influence cancer metastasis based on the core PCP pathway in breast cancer cells, indicating that PCP components are almost mutually distributed in the protrusions of single, motile and malignant cells. Exosome activity is associated with the Wnt11 produced in breast cancer cells, and exosomes secreted from fibroblast are internalized by BCCs and further loaded with Wnt11. Therefore, exosomes secreted from fibroblasts play an important role in mediating the mobilization of autocrine Wnt-PCP signaling in BCCs, stimulating invasive behavior and metastasis in murine models [21].

In a recent study, cancer-associated fibroblasts demonstrated enhanced exosome production following gemcitabine injection, which also influenced exosome content by an increase in the presence of SNAIL1 and miR-146a. After treating pancreatic cancer cells with gemcitabine-derived CAF exosomes, cancer cells showed resistance to therapy and increased proliferation. Such results emphasize the ability of stromal cell-derived exosomes to enhance pro-cancer properties, including migration and resistance to therapy [22].

## 3. Exosomes and Colon Cancer—Reported Associations

Exosomes are often employed as a novel reservoir for disease biomarker discovery, especially in cancers. There have been reports showing the usefulness of exosomal miRNA-103, tripartite motif-containing 3 protein, glypican-1 proteoglycan protein and hepatocyte growth factor-regulated tyrosine kinase substrate protein in colon cancers. As a result, exosomes proved their potential as tumor markers for various types of cancers, including colorectal cancer [23,24,25].

As cancer cells secrete more exosomes than normal cells, there is a significant difference between molecules found in tumor cell-derived exosomes and those in normal cells. It has been demonstrated that there is a significant difference in certain miRNAs, lncRNAs and proteins in blood-derived exosomes between patients with colorectal patients and healthy subjects. Such exosomal molecules could be used as predictors for colorectal cancer [24].

The serum expression level of exosomal miRNA (let-7a, miR-1229, miR-1246, miR-150, miR-21, miR-223, and miR-23a) was significantly higher in primary colorectal cancer patients, including those with early-stage disease than in healthy subjects, being substantially downregulated following tumor excision. Those seven miRNAs also showed significantly higher secretion by colon cancer cell lines when compared to the healthy colon-derived cell line.

Their high sensitivity was validated by receiver operating characteristic (ROC) analysis [26].

### Exosome and CRC Metastasis

It has been stated that 90% of cancer deaths are caused by metastasis. Commonly, colorectal cancer spreads to distant organs (liver, lung, lymph nodes). In the case of patients with distant metastasis, the five-year survival rate is a grim 10%. It is thus very important to detect metastasis early in order to increase the survival of these patients [24].

MiR-203 demonstrated the existence of a link between tumor and host cells, with exosomal miR-203 presented as a novel biomarker to predict metastasis mainly as a promotor of monocyte differentiation to M2-TAMs and the subsequent formation of pre-metastatic niches. There have been significant clinical findings showing the dual functions of miR-203 in the progression of colorectal cancer [27].

Enhanced IRF-2 serum levels in CRC patients with lymph node metastasis present themselves as a novel biomarker for metastasis. Exosomal IRF-2 is able to activate lymph node metastases by remodeling the lymphatic network [28].

Shao et al. demonstrated that serum extracellular vesicles containing miR-21 in colon cancer cells are new macrophage regulators leading to the creation of an inflammatory pre-metastatic niche in colon cancer liver metastasis. While cancer develops, primary CRC cells secrete serum extracellular vesicles containing miR-21 that are transported by the blood flow to the liver where they are engulfed by macrophages. The serum extracellular vesicles deliver the miR-21 load, and by targeting the TLR7 pathway, they polarize macrophages enhancing the synthesis and release of pro-inflammatory cytokines such as IL-6, thus paving the way for a permissive inflammatory pre-metastatic niche in the liver where circulating CRC cells can survive, colonize and subsequently develop macrometastasis [29].

Recent research has shown that miR-375 controls the expression of MMP2 and other genes involved in the epithelial–mesenchymal transition (EMT), such as SNAIL. Colorectal cancer cells proliferate, invade, and migrate when miR-375 is suppressed. Loss of function of the tumor suppressor miR-374 in colorectal cancer (CRC) promotes proliferation, invasion, migration, and intrahepatic metastasis through activation of the PIK3/AKT pathway. To a large extent, miR-374 inhibition upregulates the expression of its targets, which include the transcription factors SNAIL, SLUG, and ZEB1 as well as NCAD and VIM [30].

The regulation of ZEB transcription factors in CRC cells is primarily mediated by two members of the miR-200 family: miR-200c and miR-429. MiR-200c inhibition of ZEB1 expression leads to EMT inactivation and decreased CRC cell invasion and migration. Because of its ability to target ONECUT2, MiR-429 could suppress cell migration and invasion, reversing TGFb’s EMT-inducing effects. MiR-429 is, however, substantially downregulated in colorectal cancer [31].

Because of its ability to target ONECUT2, MiR-429 could suppress cell migration and invasion, reversing TGFb’s EMT-inducing effects. In contrast, miR-429 is considerably downregulated in colorectal cancer [32].

In addition, the loss of ASCL2 function, a target of WNT signaling, can activate the miR-200 cluster, which in turn inhibits the ZEB and SNAIL families of transcription factors and controls the plasticity from EMT to mesenchymal–epithelial transition (MET) [33].

It has been found that the upregulation of the ZEB2 target gene is associated with CRC invasion and metastasis when other tumor suppressors, particularly miR-335, miR-132, and miR-192, are downregulated [34,35,36].

Takano et al. stated that CRC cell-derived exosomal miR-203 promotes the differentiation of monocytes into M2-tumor-associated macrophages (TAMs) involved in colorectal cancer metastasis to the liver [27].

Table 1 summarizes the roles of exosomes in colorectal metastatic disease. One can distinguish the important clinical aspects in which exosomes are involved as well as opposite effects (anticancer/cancer promoter) reported for different exosomes.

## 4. Exosomal Elements as Predictive Markers for Colon Cancer

Efforts have been made to employ miRNAs in serum or plasma as diagnostic biomarkers for more cancers. There are still decisions to be made regarding the type of miRNAs to be selected as markers. The particular properties of exosomes, such as their ability to embed specific miRNAs, their stability in the blood flow, their reproducible detection, and especially their ability to reflect the properties of cancer cells, promote them as important tools in the design of highly sensitive diagnostic strategies for the rapid and non-invasive monitoring of cancer evolution [26].

Exosomal miRNAs could be a biomarker of colorectal cancer. A recent RNA sequencing study on exosomes in colorectal cancer patients indicated high miRNA-139-3p, let-7b-3p and miRNA-145-3p expression in plasma exosomes [37].

Elevated exosomal miRNA-19a levels in the serum of colorectal patients were indicative of cancer recurrence [38].

Moreover, exosomal miRNA-17-92a expression in the blood was associated with cancer recurrence. Certain exosomal miRNAs such as miRNA-1229, miRNA-1246, miRNA-21, miRNA-23a, let-7a, miRNA-223 and miRNA-150 demonstrated great transfer by serum exosomes in colorectal cancer patients, but they were significantly lower following surgical excision [26].

MiRNA-1246, miRNA-21 and miRNA-23a stand out as powerful diagnostic biomarkers of colorectal cancer [39].

**Figure 2:**
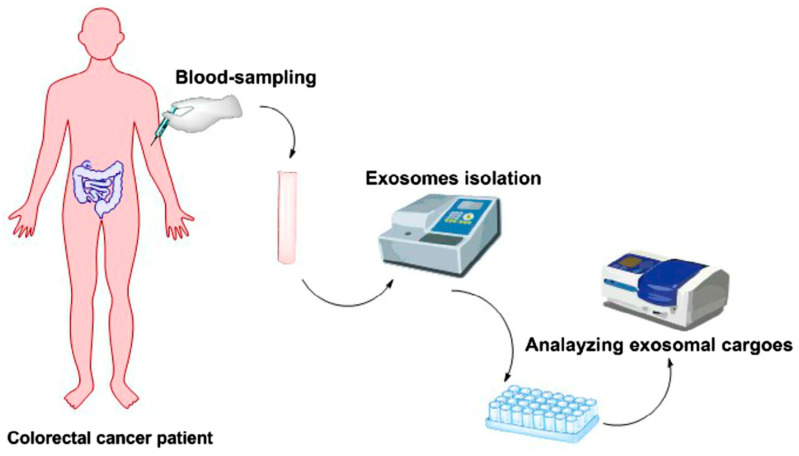
Method for highlighting types of biomarkers of colorectal cancer patient. Reprinted with permission from Ahmadi, M et al., 2021 [40].

Figure 2 illustrates one method to be implemented in the future to analyze cargoes of exosomes in order to highlight different types of miRNA embedded as biomarkers for colorectal cancer.

Table 2 shows the types of exosomal miRNAs that are potential cancer diagnostic biomarkers in colorectal cancer. The studies discussed the use of lncRNA-loaded CRC-derived exosomes as diagnostic biomarkers.

In another study, Zou et al. observed significantly lower serum exosomal miR-150-5p levels in colorectal cancer patients, therefore being appropriate diagnosis indicators. Diagnostic accuracy was boosted by the combined use of miR-150-5p and the carcinoembryonic antigen. Altogether, these findings emphasize the potential of exosomal miRNAs in diagnosing colorectal cancer [41].

LncRNA is a non-coding RNA that has a size of more than 200 nt in length, and it was found in the blood exosomes of patients diagnosed with colorectal cancer. The results in one study showed an overexpression of lncRNA differentially expressed. This could lead in using lncRNA as a tumor marker due to its non-invasive character, high sensitivity and specificity, as well as stability. It is also highly correlated with aggressive tumor behavior and poor prognosis. Such results provide the grounds for the design of an early diagnostic and prognostic biomarker for colorectal cancer and the corresponding novel therapeutic strategies [42].

In their study, Hu et al. study demonstrated that exosomal lncRNAs, namely LNCV6_98602, LNCV6_98390, LNCV_108266, LNCV6_116109, LNCV6_38772, and LNCV6_84003 plasma expression, was significantly higher in patients with colorectal cancer, promoting them as potential diagnostic biomarkers for this type of cancer [43].

Barbagallo et al. showed in two types of CRC cell lines (HCT-116, Caco-2) that urothelial cancer associated 1 (UCA1), also a lncRNA, can act as a RNA regulator for colorectal cancer progression by modulating the ceRNA network, thus upregulating ANLN, BIRC5, IPO7, KIF2A and KIF23 in two ways: (1) miRNAs sponge effects determining negative expression, and (2) the direct binding of mRNAs to 3′-UTRs to protect them from degradation. Such elaborate RNA-based regulatory signaling for cancer control suggests the design of novel anticancer therapies targeting UCA1 [44].

Granulocytic myeloid-derived suppressor cells were shown to enhance the capability of colorectal cancer cells for self-renewal and differentiation as a result of exosomes and exosomal S100A9 influence in the tumor microenvironment, mainly under hypoxic conditions. Hyperoxia reduces the stemness of colon cancer cells via the inhibition of the production of GM-Exo. Elevated plasma concentration of exosomal S100A9 was linked to the occurrence and recurrence of colorectal cancer. The production of block MDSC exosomes could be used as a new approach for colorectal therapy [45].

The results demonstrate the potential use of exosomal proteins as biomarkers of colorectal cancer.

## 5. Detection and Screening Based on Exosomal Components

The carcinoembryonic antigen (CEA) was also observed in the serum exosomes of colorectal patients [46]. The value of the area under the curve (AUC) of serum exosomal CEA (0.9354) was greater than that of serum CEA (0.8557). It is thus more significant to detect serum exosomal CEA in order to predict distant metastasis in colorectal cancer. The overexpression of interferon regulatory factor 2 (IRF-2) was observed in the serum exosomes of colorectal cancer patients with lymph node metastasis [28].

From a mechanistic view, exosomal IRF-2 triggers lymph node metastasis by remodeling the lymphatic network. Certain miRNAs were differentially expressed in the plasma exosomes of patients with locally advanced rectal cancer, therefore promoting themselves as potential biomarkers for the poor prognosis of colorectal cancer [47].

Among them, there was a correlation between low miR-181a-5p levels and high miR-30d-5p levels in plasma exosomes and lymph node metastases and liver metastases. There is still no clear definition of the roles these RNAs play in colorectal cancer [24].

In their study, Jun et al. were able to identify several candidate targets with a miRNA–mRNA network (mRNA: CBFB, CDH3, ETV4, FOXQ1, FUT1, GCNT2, GRIN2D, KIAA1549, KRT80, LZTS1, SLC39A10, SPTBN2, ZSWIM4; and exosomal miRNA: hsa-miR-126, hsa-miR-139, hsa-miR-141, hsa-miR-29c, and hsa-miR-423), which could be used as potential biomarkers in the diagnosis of colorectal cancer with the presence of an exosomal miRNA–mRNA network in cancer progression. Their results pave the way for new diagnostic and treatment strategies of colorectal cancer [48].

## 6. Targeting Exosomal Components—Drugs, Nanostructures, Polymers

There have been great attempts to enhance the innate properties of exosomes and to enhance the manufacturing process of exosomes or exosome mimetics. Exosome-based drug delivery tools were divided into three subgroups based on the extent of human manipulation and their natural feel compared with cell-derived exosomes. Frequent protein components that exosomes contain include cytoskeletal (such as actin), cytosolic (for example GAPDH), heat shock (HSP90), antigen presentation (MHC-I, -II), and membrane proteins (CD9, CD63) together with proteins involved in vesicle trafficking (Tsg101) [49].

In the tumor microenvironment (TME), fibroblasts are a major component. MicroRNAs regulate multiple signaling pathways, causing fibroblasts at the primary tumor site to take on a new phenotype and transform into CAFs. Cancer-associated fibroblasts (CAFs) are distinct from normal fibroblasts (NFs) due to their pro-tumorigenic properties and high expression of smooth muscle actin (28). To promote tumor growth, CAFs secrete a variety of pro-inflammatory molecules, such as interleukins, chemokines, and extracellular matrix (ECM) components.

Oxaliplatin (Oxa) is a common chemotherapeutic agent for colorectal cancer treatment. The exosome-mediated crosstalk between CRC-associated fibroblasts (CAFs) and CRC cells have demonstrated important roles in chemoresistance to Oxa. It was also confirmed that oncogene miR-21, one of the most oncogenic miRNAs, was enriched in the exosomes from CAFs [50].

After overexpression in exosomes, miR-21 is transported to colorectal cancer cells and enhances AKT phosphorylation strongly related to chemoresistance to Oxa. In another study, lncRNA H19 was expressed to a great extent in the CAFs of colorectal cancer patients, which also increased with cancer progression. LncRNA H19, as a oncofetal transcript, has been shown to promote SIRT1-mediated autophagy in colorectal cancer (CRC) cells, which in turn confers resistance to 5-fluorouracil [51].

One of the most common causes of therapeutic failure is resistance to therapy. The various mechanisms of exomes were shown to determine drug resistance in several recent studies. Exosomes can guide miRNAs, lncRNAs and proteins to the target cells and trigger signal transmission between drug-resistant cells and sensitive cells, stromal cells and tumor cells, which can lead to the drug resistance of tumor cells [52,53].

Other examples of resistance were observed in the microenvironment of ovarian cancer, where exosomes derived from tumor-associated adipocytes and tumor-associated fibroblasts are able to transport miR-21 to ovarian cells, downregulating APAF1 expression and inhibiting tumor apoptosis, thus leading to resistance to paclitaxel [54].

In colorectal cancer, CAF-derived exosomes loaded with miR-92a-3p are aimed at FBXW7 and MOAP1 in the tumor microenvironment and further activate the WNT/*β*-catenin pathway, inhibit mitochondrial apoptosis, leading to cell stemness, epithelial–mesenchymal transition, tumor metastasis and resistance to 5-FU/L-OHP [55].

Tumors are able to stray from attacks from the immune system by various mechanisms that allow them to avoid being detected. The immunomodulatory potential makes exosomes useful in novel immunological strategies to improve antitumor immunity.

Cancer immunotherapy using chimeric antigen receptor (CAR) is a promising therapeutic approach. The clinical use of CAR-modified T cell (CAR-T) therapy in solid tumors was not as successful as in hematological malignancies, such as acute lymphoid leukemia, mainly due to side effects such as cytokine release syndrome (CRS), cytokine storm and on-target/off-tumor responses [56].

## 7. Future Directions

Because of their notable accuracy across a wide range of biological datasets, microRNAs have emerged as promising leads in the search for additional CRC cancer biomarkers. The value of serum miRNAs throughout CRC diagnosis, prognosis, and treatment response has been the subject of a plethora of recent papers. Compared to traditional markers such as carcinoembryonic antigen (CEA) and CA19-9, a panel of six miRNAs (miR-21, let-7g, miR-31, miR-92a, miR-181b, and miR-203) has been shown to be a potential marker for CRC diagnosis with over 40% specificity and sensitivity [57].

The absence of trustworthy methods for cancer detection has led to a drawback in the development of colorectal cancer. There is a need for highly efficient detection techniques in order to lower the risk of cancer-associated mortality. More and more findings have demonstrated the strong correlation between the initiation and progression of colorectal cancer and the differentially expressed exosomal RNAs and proteins. These molecules are able to influence the oncogenesis, metastasis, chemoresistance and recurrence of colorectal cancer, thus being potential candidates for this type of cancer. There are several advantages offered by exosomes as novel tumor markers: (i) they could be superior to conventional techniques in terms of sensitivity and specificity; (ii) their bioactive molecular content, without much serum involvement; (iii) they are characterized by high stability and their contents do not degrade in the extracellular environment; and (iv) they are secreted by a variety of body liquids, and thus, they can be extracted in a non-invasive manner. The Food and Drug Administration has already approved the use of certain exosome-based diagnostic kits in clinical trials [24].

Nevertheless, there are certain drawbacks to the use of exosomes as tumor markers. For example, it is essential to rapidly and meticulously isolate exosomes from a sample prior to using them as biomarkers. The present isolation techniques have their own limitations, being bulky, lengthy, including contaminations, and they are expensive. The purity of exosomes is of great importance and the presence of impurities, such as proteins and RNAs in exosomal compounds has been reported, which may have a negative impact on the accuracy of exosome-based diagnosis. It is thus crucial to design highly accurate separation methods to enable the transition of exosome detection to clinical applications. Another aspect is the term “exosomes” itself, which is not recommended nomenclature anymore due to the wide vesicle heterogeneity depending on the purification method, which has dictated the quality and accuracy of the final product. The different results have led to standardization issues, and thus, studies cannot be compared. It is necessary to eliminate all deviations in order to successfully employ exosomes as biomarkers [24].

There are multiple mechanisms by which exosomes act as mediators of intercellular communication. Those derived from colorectal cancer cells are essential mediators in this type of cancer influencing tumor formation by enhancing growth, invasion, metastasis, angiogenesis and immunomodulation. Regardless the stage of the condition, exosomes can transport certain biomolecules into the blood, therefore promoting themselves as promising biomarkers for cancer stage. As exosomes are released into various biofluids, they could be used as a novel diagnostic biomarker in colorectal cancer. There are still a few aspects that need to be thoroughly explained, such as the processes of separation, characterization and validation [40].

## 8. Conclusions

Several studies have investigated the potential of exosomes as diagnostic and prognostic biomarkers in cancer in general and colorectal cancer in specific and as targets for novel therapeutic interventions. However, further research is needed to fully understand the complex roles of exosomes in colorectal cancer and to translate this knowledge into clinical practice. Overall, exosomes represent a promising area of investigation for improving the diagnosis, treatment, and management of colorectal cancer.

It has become increasingly evident that there are several key aspects regarding the underlying mechanisms of exosome-mediated crosstalk in the tumor microenvironment, distant cell interactions, exosome heterogeneity, and molecular mechanisms that are responsible for resistance and metastasis. Our understanding of exosome-mediated therapy-resistance in different cancers will be directed by the tumor context, which will be directed by the design of different research approaches in this new vast area of study based on the tumor context. The translation of these findings into the clinical realm will provide a novel and effective treatment modality for future cancer patients.

Due to their quantity and heterogeneity, exosome biomarkers can produce false positives and negatives in diagnosis and prognosis. Clinical exosome biomarker sensitivity and specificity must be improved.

Blocking the formation of pre-metastatic niches and inhibiting miRNAs intracellular signaling to prevent metastasis may be used as a novel cancer therapy strategy.

## Figures and Tables

**Table 1:** Exosomes in colorectal metastatic disease.

Name	Role	Clinical Implication
miR-203	promotor of monocyte differentiation to M2-TAMs	carcinogenesis and progression by promoting tumor growth, proliferation, antiapoptotic mechanisms, and migration [27]
IRF-2	vascular endothelial growth factor C	activate lymph node metastases by remodeling the lymphatic network [28]
miR-21	TLR7 pathway	polarize macrophages enhancing synthesis and release of pro-inflammatory cytokines such as IL-6 [29]
miR-375	controls expression of MMP2 (and other genes involved in the epithelial-mesenchymal transition (EMT)), SNAIL gene	promotes proliferation, invasion, migration, and intrahepatic metastasis through activation of the PIK3/AKT pathway [30]
miR-200c	inhibition of ZEB1 expression	EMT inactivation and decreased CRC cell invasion and migration [31]
miR-429	target ONECUT2	suppress cell migration and invasion [32]
miR-335 miR-132 miR-192	upregulation of the ZEB2	CRC invasion and metastasis [27]

**Table 2:** Potential cancer diagnostic biomarkers miRNA in colorectal cancer. Adapted with permission from Ahmadi, M et al., 2021 [40].

miRNA Type	Isolated from	Level of miRNA in CRC
mrRNA-23a; miRNA-301a	Serum	High
miRNA-486-5p	Plasma	High
miRNA-6803-5p	Serum	High
miRNA-125a-3p	Plasma	High
miRNA-150-5p	Serum	Low

## Data Availability

Not applicable.

## References

[1] De Toro J., Herschlik L., Waldner C., Mongini C. (2015). Emerging roles of exosomes in normal and pathological conditions: New insights for diagnosis and therapeutic applications. Front. Immunol..

[2] Ruivo C.F., Adem B., Silva M., Melo S.A. (2017). The Biology of Cancer Exosomes: Insights and New PerspectivesBiology of Cancer Exosomes. Cancer Res..

[3] Zhang H., Lu J., Liu J., Zhang G., Lu A. (2020). Advances in the discovery of exosome inhibitors in cancer. J. Enzym. Inhib. Med. Chem..

[4] Tkach M., Théry C. (2016). Communication by extracellular vesicles: Where we are and where we need to go. Cell.

[5] Zhang L., Yu D. (2019). Exosomes in cancer development, metastasis, and immunity. Biochim. Biophys. Acta BBA Rev. Cancer.

[6] Kowal J., Arras G., Colombo M., Jouve M., Morath J.P., Primdal-Bengtson B., Dingli F., Loew D., Tkach M., Théry C. (2016). Proteomic comparison defines novel markers to characterize heterogeneous populations of extracellular vesicle subtypes. Proc. Natl. Acad. Sci. USA.

[7] Zhu L., Sun H., Wang S., Huang S., Zheng Y., Wang C., Hu B., Qin W., Zou T., Fu Y. (2020). Isolation and characterization of exosomes for cancer research. J. Hematol. Oncol..

[8] Robbins P.D., Morelli A.E. (2014). Regulation of immune responses by extracellular vesicles. Nat. Rev. Immunol..

[9] Raposo G., Nijman H.W., Stoorvogel W., Liejendekker R., Harding C.V., Melief C.J., Geuze H.J. (1996). B lymphocytes secrete antigen-presenting vesicles. J. Exp. Med..

[10] Théry C., Duban L., Segura E., Véron P., Lantz O., Amigorena S. (2002). Indirect activation of naïve CD4 T cells by dendritic cell–derived exosomes. Nat. Immunol..

[11] Wolfers J., Lozier A., Raposo G., Regnault A., Théry C., Masurier C., Flament C., Pouzieux S., Faure F., Tursz T. (2001). Tumor-derived exosomes are a source of shared tumor rejection antigens for CTL cross-priming. Nat. Med..

[12] Vega V.L., Rodriguez-Silva M., Frey T., Gehrmann M., Diaz J.C., Steinem C., Multhoff G., Arispe N., De Maio A. (2008). Hsp70 translocates into the plasma membrane after stress and is released into the extracellular environment in a membrane-associated form that activates macrophages. J. Immunol..

[13] Benson M.J., Dillon S.R., Castigli E., Geha R.S., Xu S., Lam K., Noelle R.J. (2008). Cutting edge: The dependence of plasma cells and independence of memory B cells on BAFF and APRIL. J. Immunol..

[14] Lv L., Wan Y., Lin Y., Zhang W., Yang M., Li G., Lin H., Shang C., Chen Y., Min J. (2012). Anticancer drugs cause release of exosomes with heat shock proteins from human hepatocellular carcinoma cells that elicit effective natural killer cell antitumor responses in vitro. J. Biol. Chem..

[15] Clayton A., Mitchell J.P., Court J., Mason M.D., Tabi Z. (2007). Human tumor-derived exosomes selectively impair lymphocyte responses to interleukin-2. Cancer Res..

[16] Yu S., Liu C., Su K., Wang J., Liu Y., Zhang L., Li C., Cong Y., Kimberly R., Grizzle W.E. (2007). Tumor exosomes inhibit differentiation of bone marrow dendritic cells. J. Immunol..

[17] Raimondo S., Saieva L., Corrado C., Fontana S., Flugy A., Rizzo A., De Leo G., Alessandro R. (2015). Chronic myeloid leukemia-derived exosomes promote tumor growth through an autocrine mechanism. Cell Commun. Signal..

[18] Kalluri R. (2016). The biology and function of exosomes in cancer. J. Clin. Investig..

[19] Choi D., Lee T.H., Spinelli C., Chennakrishnaiah S., D’Asti E., Rak J. (2017). Extracellular Vesicle Communication Pathways as Regulatory Targets of Oncogenic Transformation, Seminars in Cell & Developmental Biology.

[20] Thierry A., El Messaoudi S., Gahan P., Anker P., Stroun M. (2016). Origins, structures, and functions of circulating DNA in oncology. Cancer Metastasis Rev..

[21] Luga V., Zhang L., Viloria-Petit A.M., Ogunjimi A.A., Inanlou M.R., Chiu E., Buchanan M., Hosein A.N., Basik M., Wrana J.L. (2012). Exosomes mediate stromal mobilization of autocrine Wnt-PCP signaling in breast cancer cell migration. Cell.

[22] Richards K.E., Zeleniak A.E., Fishel M.L., Wu J., Littlepage L.E., Hill R. (2017). Cancer-associated fibroblast exosomes regulate survival and proliferation of pancreatic cancer cells. Oncogene.

[23] Sun Y., Zheng W., Guo Z., Ju Q., Zhu L., Gao J., Zhou L., Liu F., Xu Y., Zhan Q. (2016). A novel TP53 pathway influences the HGS-mediated exosome formation in colorectal cancer. Sci. Rep..

[24] Xiao Y., Zhong J., Zhong B., Huang J., Jiang L., Jiang Y., Yuan J., Sun J., Dai L., Yang C. (2020). Exosomes as potential sources of biomarkers in colorectal cancer. Cancer Lett..

[25] Lu F., Chen S., Shi W., Su X., Wu H., Liu M. (2022). GPC1 promotes the growth and migration of colorectal cancer cells through regulating the TGF-β1/SMAD2 signaling pathway. PLoS ONE.

[26] Ogata-Kawata H., Izumiya M., Kurioka D., Honma Y., Yamada Y., Furuta K., Gunji T., Ohta H., Okamoto H., Sonoda H. (2014). Circulating exosomal microRNAs as biomarkers of colon cancer. PLoS ONE.

[27] Takano Y., Masuda T., Iinuma H., Yamaguchi R., Sato K., Tobo T., Hirata H., Kuroda Y., Nambara S., Hayashi N. (2017). Circulating exosomal microRNA-203 is associated with metastasis possibly via inducing tumor-associated macrophages in colorectal cancer. Oncotarget.

[28] Sun B., Zhou Y., Fang Y., Li Z., Gu X., Xiang J. (2019). Colorectal cancer exosomes induce lymphatic network remodeling in lymph nodes. Int. J. Cancer.

[29] Shao Y., Chen T., Zheng X., Yang S., Xu K., Chen X., Xu F., Wang L., Shen Y., Wang T. (2018). Colorectal cancer-derived small extracellular vesicles establish an inflammatory premetastatic niche in liver metastasis. Carcinogenesis.

[30] Chen Y., Jiang J., Zhao M., Luo X., Liang Z., Zhen Y., Fu Q., Deng X., Lin X., Li L. (2016). microRNA-374a suppresses colon cancer progression by directly reducing CCND1 to inactivate the PI3K/AKT pathway. Oncotarget.

[31] Hur K., Toiyama Y., Takahashi M., Balaguer F., Nagasaka T., Koike J., Hemmi H., Koi M., Boland C.R., Goel A. (2013). MicroRNA-200c modulates epithelial-to-mesenchymal transition (EMT) in human colorectal cancer metastasis. Gut.

[32] Sun Y., Shen S., Liu X., Tang H., Wang Z., Yu Z., Li X., Wu M. (2014). MiR-429 inhibits cells growth and invasion and regulates EMT-related marker genes by targeting Onecut2 in colorectal carcinoma. Mol. Cell. Biochem..

[33] Tian Y., Pan Q., Shang Y., Zhu R., Ye J., Liu Y., Zhong X., Li S., He Y., Chen L. (2014). MicroRNA-200 (miR-200) cluster regulation by achaete scute-like 2 (Ascl2): Impact on the epithelial-mesenchymal transition in colon cancer cells. J. Biol. Chem..

[34] Sun Z., Zhang Z., Liu Z., Qiu B., Liu K., Dong G. (2014). MicroRNA-335 inhibits invasion and metastasis of colorectal cancer by targeting ZEB2. Med. Oncol..

[35] Zheng Y.B., Luo H.P., Shi Q., Hao Z.N., Ding Y., Wang Q.S., Li S.B., Xiao G.C., Tong S.L. (2014). miR-132 inhibits colorectal cancer invasion and metastasis via directly targeting ZEB2. World J. Gastroenterol..

[36] Geng L., Chaudhuri A., Talmon G., Wisecarver J.L., Are C., Brattain M., Wang J. (2014). MicroRNA-192 suppresses liver metastasis of colon cancer. Oncogene.

[37] Min L., Zhu S., Chen L., Liu X., Wei R., Zhao L., Yang Y., Zhang Z., Kong G., Li P. (2019). Evaluation of circulating small extracellular vesicles derived miRNAs as biomarkers of early colon cancer: A comparison with plasma total miRNAs. J. Extracell. Vesicles.

[38] Matsumura T., Sugimachi K., Iinuma H., Takahashi Y., Kurashige J., Sawada G., Ueda M., Uchi R., Ueo H., Takano Y. (2015). Exosomal microRNA in serum is a novel biomarker of recurrence in human colorectal cancer. Br. J. Cancer.

[39] Lalkhen A.G., McCluskey A. (2008). Clinical tests: Sensitivity and specificity. Contin. Educ. Anaesth. Crit. Care Pain.

[40] Ahmadi M., Jafari R., Mahmoodi M., Rezaie J. (2021). The tumorigenic and therapeutic functions of exosomes in colorectal cancer: Opportunity and challenges. Cell Biochem. Funct..

[41] Zou S., Chen Y., Ge Z., Qu Y., Cao Y., Kang Z. (2019). Downregulation of serum exosomal miR-150-5p is associated with poor prognosis in patients with colorectal cancer. Cancer Biomark..

[42] Liu T., Zhang X., Gao S., Jing F., Yang Y., Du L., Zheng G., Li P., Li C., Wang C. (2016). Exosomal long noncoding RNA CRNDE-h as a novel serum-based biomarker for diagnosis and prognosis of colorectal cancer. Oncotarget.

[43] Hu D., Zhan Y., Zhu K., Bai M., Han J., Si Y., Zhang H., Kong D. (2018). Plasma Exosomal Long Non-Coding RNAs Serve as Biomarkers for Early Detection of Colorectal Cancer. Cell. Physiol. Biochem..

[44] Barbagallo C., Brex D., Caponnetto A., Cirnigliaro M., Scalia M., Magnano A., Caltabiano R., Barbagallo D., Biondi A., Cappellani A. (2018). LncRNA UCA1, upregulated in CRC biopsies and downregulated in serum exosomes, controls mRNA expression by RNA-RNA interactions. Mol. Ther. Nucleic Acids.

[45] Wang Y., Yin K., Tian J., Xia X., Ma J., Tang X., Xu H., Wang S. (2019). Granulocytic Myeloid-Derived suppressor cells promote the stemness of colorectal cancer cells through exosomal S100A9. Adv. Sci..

[46] Yokoyama S., Takeuchi A., Yamaguchi S., Mitani Y., Watanabe T., Matsuda K., Hotta T., Shively J.E., Yamaue H. (2017). Clinical implications of carcinoembryonic antigen distribution in serum exosomal fraction—Measurement by ELISA. PLoS ONE.

[47] Bjørnetrø T., Redalen K.R., Meltzer S., Thusyanthan N.S., Samiappan R., Jegerschöld C., Handeland K.R., Ree A.H. (2019). An experimental strategy unveiling exosomal microRNAs 486-5p, 181a-5p and 30d-5p from hypoxic tumour cells as circulating indicators of high-risk rectal cancer. J. Extracell. Vesicles.

[48] Ma J., Wang P., Huang L., Qiao J., Li J. (2021). Bioinformatic analysis reveals an exosomal miRNA-mRNA network in colorectal cancer. BMC Med. Genom..

[49] Shao J., Zaro J., Shen Y. (2020). Advances in Exosome-Based Drug Delivery and Tumor Targeting: From Tissue Distribution to Intracellular Fate. Int. J. Nanomed..

[50] Bhome R., Goh R.W., Bullock M.D., Pillar N., Thirdborough S.M., Mellone M., Mirnezami R., Galea D., Veselkov K., Gu Q. (2017). Exosomal microRNAs derived from colorectal cancer-associated fibroblasts: Role in driving cancer progression. Aging.

[51] Ren J., Ding L., Zhang D., Shi G., Xu Q., Shen S., Wang Y., Wang T., Hou Y. (2018). Carcinoma-associated fibroblasts promote the stemness and chemoresistance of colorectal cancer by transferring exosomal lncRNA H19. Theranostics.

[52] Shedden K., Xie X.T., Chandaroy P., Chang Y.T., Rosania G.R. (2003). Expulsion of small molecules in vesicles shed by cancer cells: Association with gene expression and chemosensitivity profiles. Cancer Res..

[53] Bach D., Hong J., Park H.J., Lee S.K. (2017). The role of exosomes and miRNAs in drug-resistance of cancer cells. Int. J. Cancer.

[54] Au Yeung C.L., Co N., Tsuruga T., Yeung T., Kwan S., Leung C.S., Li Y., Lu E.S., Kwan K., Wong K. (2016). Exosomal transfer of stroma-derived miR21 confers paclitaxel resistance in ovarian cancer cells through targeting APAF1. Nat. Commun..

[55] Hu J., Wang W., Lan X., Zeng Z., Liang Y., Yan Y., Song F., Wang F., Zhu X., Liao W. (2019). CAFs secreted exosomes promote metastasis and chemotherapy resistance by enhancing cell stemness and epithelial-mesenchymal transition in colorectal cancer. Mol. Cancer.

[56] Wang Z., Wu Z., Liu Y., Han W. (2017). New development in CAR-T cell therapy. J. Hematol. Oncol..

[57] Balacescu O., Sur D., Cainap C., Visan S., Cruceriu D., Manzat-Saplacan R., Muresan M.-S., Balacescu L., Lisencu C., Irimie A. (2018). The Impact of miRNA in Colorectal Cancer Progression and Its Liver Metastases. Int. J. Mol. Sci..

